# Location influences snacking behavior of US infants, toddlers and preschool children

**DOI:** 10.1186/s12889-018-5576-5

**Published:** 2018-06-13

**Authors:** Emma F. Jacquier, Denise M. Deming, Alison L. Eldridge

**Affiliations:** 1 Nestlé Research Center, Vers-Chez-les-Blanc, Case postale 44, 1000 Lausanne, Vaud Switzerland; 2 Nestlé Nutrition, 12 Vreeland Rd, Florham Park, New Jersey, NJ 07962 USA

**Keywords:** Snacking, Infants, Child, Preschool, Eating behavior

## Abstract

**Background:**

Compare at-home and away-from-home snacking patterns of US infants and young children.

**Methods:**

A secondary analysis was conducted using nationwide, cross-sectional dietary survey data from the US Feeding Infants and Toddlers Study (FITS) 2008. The sample included infants (6–11.9 months, *n* = 505), toddlers (12–23.9 months, *n* = 925), preschool children (24–47.9 months, *n* = 1461). Weighted population descriptive statistics (means and standard errors) were calculated using SAS. Significance was determined at *P* ≤ 0.05. The main outcome measures of the analyses were the percent of children consuming snacks by location (at home, away from home) and snacking period (morning, afternoon and evening), energy and food groups consumed during snacks.

**Results:**

Snacking at home was more prevalent than snacking away from home (toddlers, 73% vs 27%; preschoolers, 67% vs 33%). Away-from-home snacks provided about 50 additional calories per day for toddlers (346 vs 298 kcal/day, *P* ≤ 0.05) and preschoolers (371 vs 326 kcal/day, *P* ≤ 0.05) versus snacks consumed at home. Caregivers made similar snack choices for toddlers and preschoolers (milk/milk products, fruit/juice, grains and sweets) but differed in frequency of consumption by location. Among toddlers, milk/milk products were the most frequently consumed snacks at home (66%), while sweets were the top snacks consumed away from home (69%). Among preschoolers, sweets were the top snacks both at home (60%) and away (83%).

**Conclusions:**

Location is an important factor influencing snacking patterns of young children and should be considered when developing feeding guidelines. This data may be of use in the upcoming development of dietary guidelines in the U.S. for the population aged 0–2 years.

## Background

The practice of snacking is of concern within the nutrition field. While very young children need small, nutrient-dense meals and snacks [1], an increased number of eating occasions has been linked to increased energy intake in children, both in the US and Mexico [2–4]. Dietary habits formed in childhood may impact later food intake behaviors, body weight outcomes [5] and longer term health [6–8]. It is important, therefore, that caregivers of babies and young children help them to develop healthy dietary behaviors very early in life. Existing data among children aged 0–23.9 months, from the Feeding Infants and Toddlers study (FITS) 2008, found that young children’s diets were low in fruits and vegetables [9] and that approximately 85% of young preschoolers consumed some type of sweet food (dessert, candy etc.), sweetened beverage or salty snack on any given day [10]. In a recent cross-sectional analysis of data from FITS 2008, Deming et al. [11] reported that snacks provided 25% of daily energy from the age of 12 months with the percentage of energy from carbohydrates eaten as snacks (28%) exceeding the percentage of daily energy from snacks (25%). In addition, total and added sugar were also higher than the percentage of daily energy from snacks (33 and 36%, respectively).

Consistent with the findings from FITS, national survey data in the US covering a 20-year period showed a marked increase in foods high in added sugars, solid fats and sodium in the preschooler diet [12]. Further data from the National Health and Nutrition Examination Survey (NHANES) 2011–12 showed that 96% of children and adolescents consumed some food or beverage (including water) between meals [13] thus showing that snacking habits continue into later childhood. The proportion of daily energy from snacks remains relatively constant into later childhood with approximately 25% of daily energy coming from snack foods and the choice of snack foods are less nutritious in older versus younger children [14, 15]. In addition, children in the US consume approximately one third of their daily energy intake outside of the home environment [16]. However, little is known about how location affects snacking behaviors and energy intake from snacks, particularly in the first few years of life. Research in older children indicates that snacking habits vary according to the time of day with the least energy consumed during the morning snacking period [17]. Energy consumed late in the day, and evening snacking, have been associated with certain health risks in children, such as obesity and the risk of developing metabolic syndrome [18, 19]. However the nature of the relationship between snacking and overweight/obesity among children and adolescents remains equivocal [20].

The objective of this study, therefore, is to examine snacking behaviors according to location (either at home, or away from home) along with the timing of snacking during morning, afternoon and evening snacking periods, for a large sample of infants, toddlers and preschool children in the US.

## Methods

The FITS 2008 survey was a cross-sectional study which estimated food and nutrient intakes along with feeding behaviors in a national sample of US infants, toddlers and young children (from birth to 47.9 months) [21]. The FITS 2008 sample was then weighted to be nationally representative of the U.S. population of children < 4 years. Parents or primary caregivers participated by completing a telephone recruitment interview followed by a 24-h dietary recall, also by telephone. The recruitment interview collected information about family demographic characteristics, child development, eating and activity patterns and parent/caregiver knowledge and attitudes about infant feeding.

Prior to the 24-h recalls, a box containing measurement aids and a portion size booklet were mailed to participants to assist in portion size estimation. The 24-h recalls were conducted using multiple-pass methodology by trained interviewers from the University of Minnesota Nutrition Coordinating Center using the Nutrition Data System for Research (NDSR 2008). If caregivers were unsure about foods and beverages consumed at day care, dietary interviewers were trained in a protocol to obtain the information. This involved, five to ten phone call attempts, email contact, offering to call the daycare provider, or, sending a form entitled “foods fed by other adults” to request the information from a provider.

A random subsample of respondents provided an additional 24-h dietary recall so that usual nutrient intakes could be estimated. Dietary intake data was obtained for 3273 children: 382 infants aged 0 to 5.9 months, 505 infants aged 6 to 11.9 months, 925 toddlers aged 12 to 23.9 months, and 1461 children aged 24–47.9 months (hereafter referred to as 6–11.9, 12–23.9 and 24–47.9 months, respectively).

Snacking was defined as any food or beverage other than breast milk or infant formula consumed outside of mealtimes. Since the focus of this research was on snacking behaviors, and infants 0–5.9 months are still predominately consuming breast milk or infant formula, we have excluded them from further analysis. Therefore, the total sample for this research is 2891 infants, toddlers and children from 6- to 47.9 months.

During the 24-h dietary recall, caregivers were asked to define the type of eating occasion (e.g. breakfast, lunch, dinner or snack) and the time and location of each eating occasion. Using the timing of the eating occasion, snacks were further allocated into morning, afternoon and evening snacking periods. The 4897 unique foods reported in FITS 2008 were then assigned to food groups in a manner consistent with those used for food group analysis in the FITS 2002 [21]. Children were then assigned to one of two snack location groups: “Snacks at home” or “Snacks away from home” (if any of the snack occasions were consumed outside of the home). Locations for snacks away from home included day care, friend’s house, restaurant, cafeteria, fast food outlet, or school. Any other places cited by participants that did not fall into the predefined locations was coded as “other”. Sample weights were used to approximate the US population demographic characteristics. Chi-Square analysis was used to examine characteristics of the study sample by age and snacking location. Analysis of variance were performed using SAS, version 9.4 (SAS Institute, Cary, NC) to examine the frequency of foods consumed as snacks, by location. Standard errors and tests of significance were performed using SUDAAN (release 9, Research Triangle Institute, Research Triangle Park, NC). We present comparisons between snack location and by snacking time-periods in terms of group means and standard errors (SE) for the percentage of children, the number of snacks, absolute calories and the percent of calories from snacks, and frequency of consumption of different food and beverage groups by location.

## Results

Snacking location was influenced by daycare attendance, mother’s education, employment and income (Table 1). Overall, snacking away from home was significantly higher among toddlers and young children attending daycare and having a mother who works; conversely, snacking at home was higher in those not attending daycare and having a mother who does not work. Participation in the Supplemental Nutrition Program for Women, Infants and Children (commonly referred to as WIC) and annual household income were not strongly related to snacking habits at home or away from home, apart from among children of preschool age. Across all age groups snacking at home was higher among Hispanic or Latino children than snacking away from home, but the opposite was true for non-Hispanic children.

**Table 1:** Characteristics of the U.S. Feeding Infants and Toddlers Study Sample, 2008, by Age Group and Snacking Location^a^

Characteristics (%)	6–11.9 months (*n* = 505)					12–23.9 months (*n* = 925)					24–47.9 months (*n* = 1461)				
	At home		Away from home		*P*-value	At home		Away from home		*P*-value	At home		Away from home		*P*-value
	Mean	SE mean	Mean	SE mean		Mean	SE mean	Mean	SE mean		Mean	SE mean	Mean	SE mean	
Male	55.9	5.3	59.3	8.9	0.7454	48.5	3.2	44.6	5.4	0.5387	56.1	2.8	53.7	3.7	0.6056
Female	44.1	5.3	40.7	8.9		51.6	3.2	55.4	5.4		43.9	2.8	46.4	3.7	
Ethnicity															
Hispanic or latino	26.6	4.7	2.6	2.1	***0.0001***	25.5	2.8	9.5	3.5	***0.0060***	25.9	2.6	14.0	2.8	***0.0074***
Non-Hispanic or latino	73.4	4.7	97.4	2.1		74.5	2.8	90.5	3.5		74.1	2.6	86.0	2.8	
Receives WIC benefits															
Yes	46.9	5.6	27.1	8.4	0.0704	34.9	3.0	32.1	5.5	0.6555	25.8	2.5	15.4	2.6	***0.0061***
No	53.1	5.6	72.9	8.4		65.1	3.0	67.9	5.5		74.2	2.5	84.6	2.6	
Attends daycare															
Yes	39.4	5.5	52.3	9.0	0.2192	39.5	3.1	61.0	5.5	***0.0011***	50.6	2.8	73.1	3.2	***0.0000***
No	60.6	5.5	47.7	9.0		60.5	3.1	39.0	5.5		49.4	2.8	26.9	3.2	
Mother’s education															
≤11th grade	1.8	1.1	2.3	2.3	0.1227	3.6	1.9	0.5	0.5	0.3154	3.0	1.1	1.2	0.8	***0.0121***
Completed high school	35.7	6.2	26.6	8.6		21.4	2.7	17.0	5.4		18.7	2.5	12.3	2.3	
Some post-secondary	23.8	4.3	19.6	7.7		36.4	3.3	33.6	5.7		30.5	2.8	22.5	3.7	
Completed college	30.2	6.7	25.3	7.6		22.3	2.8	26.3	4.4		32.6	2.9	38.3	4.0	
Graduate work/degree	8.5	2.5	26.2	7.8		16.3	2.1	22.7	5.4		15.2	2.2	25.7	4.0	
Mother works															
Yes	54.5	5.9	60.3	9.3	0.6032	45.6	3.2	66.0	5.2	***0.0007***	51.4	3.1	61.9	4.0	***0.0375***
No	45.5	5.9	39.7	9.3		54.4	3.2	34.0	5.2		48.7	3.1	38.1	4.0	
Annual household income															
<$10,000	10.7	4.3	2.6	2.5	0.2988	5.7	1.1	4.8	2.4	0.2242	4.9	1.2	2.9	1.3	***0.0030***
$10,000–$19,999	8.6	2.7	6.7	4.6		5.6	1.2	1.7	0.8		12.0	2.3	4.7	1.7	
$20,000–$34,000	14.7	3.8	11.0	7.5		18.0	2.6	15.1	3.8		15.8	2.3	6.3	1.5	
$35,000–$49,999	18.2	4.1	10.3	4.8		19.4	2.7	15.7	4.6		15.0	1.9	13.3	2.4	
$50,000–$74,000	29.1	6.1	29.8	8.1		20.0	2.2	24.1	4.2		19.0	2.0	26.0	3.5	
$75,000–$99,999	10.0	3.1	16.2	6.2		16.3	2.7	16.9	4.2		16.1	2.2	21.9	3.3	
$100,000–$149,000	6.6	2.3	18.9	8.7		9.2	1.8	18.0	4.4		11.5	1.7	16.5	3.3	
>$150,000	2.2	1.0	4.6	2.7		5.9	2.1	3.7	1.2		5.8	1.4	8.4	2.8	
All Snacks Sample Size	221		53			645		233			929		466		

*P*-Values in bold italics indicates results are statistically significant at *p* ≤ 0.05

^a^Table 1 was developed using Chi-Square analysis using SAS v 9.4 software

### Snacking location and snacking periods

Across all age groups, infants and young children consumed more snacks at home than away from home, but as age increased, the percent consuming snacks away from home increased (Table 2). Only about half of infants (54.3%) consumed foods and beverages other than breast milk or infant formula as snacks. Snacks were consumed by 94.9% 12–23.9 months of age and by 95.5% of children 24–47.9 months. Among infants who consumed snacks, nearly all consumed morning snacks (91.3%) and evening snacks (95.7%) at home. About 20% of infants consumed the afternoon snack away from home. There was an increase in snacking away from home in toddlers (12–23.9 months), especially during the morning period (24.8%, compared to 8.7% among infants). Among preschool children (24–47.9 months), 28.3% ate the morning snack away from home, and 25.5% ate the afternoon snack away.

**Table 2:** Number and Proportion of Snacks Consumed during Different Snacking Periods at Home and Away from Home by U.S. Children in the Feeding Infants and Toddlers study, 2008^a^

Snacking period	6–11.9 months (*n* = 505)				12–23.9 months (*n* = 925)				24–47.9 months (*n* = 1461)			
	At home		Away from home		At home		Away from home		At home		Away from home	
	*n*	% (SE)	*n*	% (SE)	*n*	% (SE)	*n*	% (SE)	*n*	% (SE)	*n*	% (SE)
Morning snack^b^	100	91.3 (3.3)	10	8.7 (3.3)	462	75.2 (3.2)	118	24.8 (3.2)	634	71.7 (2.5)	255	28.3 (2.5)
Afternoon snack^b^	148	80.7 (3.6)	45	19.3 (3.6)	597	79.5 (2.2)	170	20.5 (2.2)	906	74.5 (2)	288	25.5 (2)
Evening snack^b^	95	95.7 (2.4)	5	4.3 (2.4)	461	94.4 (1.6)	25	5.6 (1.6)	788	91.5 (1.7)	60	8.5 (1.7)
All snacks^b^	221	84.9 (2.6)	53	15.1 (2.6)	645	72.7 (2.5)	233	27.3 (2.5)	929	67.3 (2.1)	466	32.7 (2.1)

^a^Away from home is any location outside of home including day care, friend’s house, restaurant, cafeteria, fast food outlet, school or other

^b^Snacks include any food or beverage consumed between meals and excludes consumption of breast milk or infant formula

### Energy and food groups consumed as snacks

**Fig. 1:**
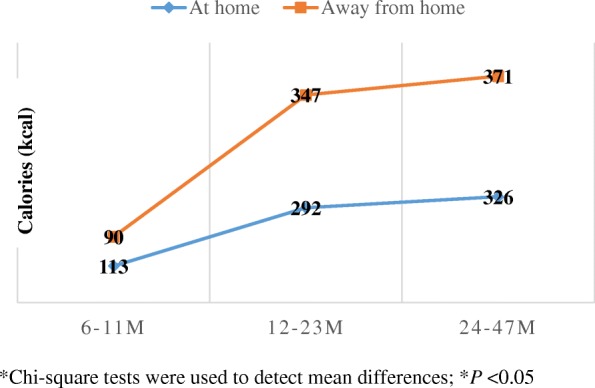
Absolute calories (kcal) from snacks consumed at home versus away from home by children in the Feeding Infants and Toddlers study 2008

The energy contribution from snacking at home and away from home was somewhat different, with lower energy contribution at home (21–25% of total daily energy) and higher energy from snacks away from home (24–28% of total energy) (Table 3). This trend towards an increased contribution to total daily energy intake from away-from-home snacking, reached significance in children 24–47.9 months of age. However, when looking at the absolute daily calories from snacks, we can see that away-from-home snacks provided significantly more energy for both toddlers and preschool children (Fig. 1) By the age of 12 months, children snacking away from home consumed about 50 kcal/day more from snacks than children snacking at home.

**Table 3:** Mean Contribution to Total Energy Intake from Snacks Consumed at Home versus Away from Home among U.S. Infants, Toddlers and Preschoolers from the Feeding Infants and Toddlers study, 2008 (*n* = 2891)

	At home		Away from home	
	*n*	Percent total daily energy from snacks	*n*	Percent total daily energy from snacks
6–12 months	230	21.2	55	23.9
12–24 months	648	26.6	234	27.6
24–48 months	929	25	466	***28.2*** ^*^

^*^Significance at *P* = 0.0145

Significant *p* values are in bold italics

We observed differences in the types of foods consumed as snacks at home and away from home (Table 4). Among 6–11.9 month olds, the categories of grain products, fruits, other foods and beverages, and sweets ranked in the same order, but a higher percentage of infants consumed grains and sweets away from home. Vegetables ranked fifth in the top five food groups consumed by infants at home, while milk and dairy ranked fifth as snacks consumed away from home. Among toddlers (12–23.9 months of age) milk and milk products consumed as snacks were most frequently consumed at home (65.6%) versus away from home (53.1%). The opposite was true for sweets where consumption was higher away from home (83.4%) versus at home (63%). By the age of 24 months sweets were the most popular choice of snack both at home (60%) and away from home (83%).

**Table 4:** Frequency of Consumption of Major Food Groups Consumed as Snacks by Location and Age Group among US Infants, Toddlers and Preschoolers from the Feeding Infants and Toddlers study, 2008^2^

At home (6–11.9 months)				Away from home (6–11.9 months)				At home (12–23.9 months)				Away from home (12–23.9 months)				At home (24–47.9 months)				Away from home (24–47.9 months)			
Major food group	Rank	*N*	Weighted % consuming	Major food group	Rank	*N*	Weighted % consuming	Major food group	Rank	*N*	Weighted % consuming	Major food group	Rank	*N*	Weighted % consuming	Major food group	Rank	*N*	Weighted % consuming	Major food group	Rank	*N*	Weighted % consuming
Grains & grain products	1	142	62.4	Grains & grain products	1	41	81.5	Milk and milk products	1	380	62.2%	Sweets, sweetened beverages and desserts	1	152	69.5%	Sweets, sweetened beverages and desserts	1	600	60.3%	Sweets, sweetened beverages and desserts	1	379	83.4%
Fruit^1^	2	92	48.8	Fruit	2	22	40.5	Fruit	2	379	56.1%	Fruit	2	143	65.6%	Milk and milk products	2	506	58.4%	Other foods and beverages	2	296	60.1%
Other foods & beverages	3	63	22.8	Other foods & beverages	3	16	29.3	Grains and grain products	3	379	54.7%	Grains and grain products	3	167	64.6%	Fruit	3	533	57.3%	Fruit	3	257	55.2%
Sweets, sweetened beverages & desserts	4	52	20.6	Sweets, sweetened beverages & desserts	4	15	27.9	Other foods and beverages	4	325	51.6%	Other foods and beverages	4	123	57.1%	Other foods and beverages	4	513	53.8%	Grains and grain products	4	267	54%
Vegetables	5	25	9.7	Milk & milk products	5	9	10.5	Sweets, sweetened beverages and desserts	5	314	49.1%	Milk and milk products	5	137	52.2%	Grains and grain products	5	430	44.4%	Milk and milk products	5	243	46.4%

^1^100% Fruit juice can be found in the major food group “Fruit”

^2^Table was developed using an analysis of variance: software SAS v9.4

## Discussion

Snacking behaviors of US infants, toddlers and preschool children varied according to location and the time of day. Snacking behaviors occurred regardless of whether the primary caregiver worked and child attended daycare or whether the primary caregiver did not work and the child stayed at home. At 12 months, almost 95% of children were consuming snacks either at home or away from home, on the day of the survey. This is consistent with the American Academy of Pediatrics guidelines which recommends 4–6 eating occasions per day for very young children - consisting of three meals and up to two snacks - in order to meet nutritional requirements [22]. Similarly, the prevalence of snacking is high among older populations of US children and adults. A recent analysis of snacking behaviors in US children aged 4–13 years found that about 96% of children reported eating at least one snack per day. As adults, data indicates that 90% of American adults report snacking on a given day [23]. The high prevalence of snacking at a young age inside and outside the home, whilst consistent with AAP recommendations to consume up to two snacks per day, also mirrors U.S. adult dietary behaviors. With approximately 90% of US adults reporting consuming at least one snack on a given day, it may follow therefore, that caregivers provide snacks to young children since they engage in snacking behavior themselves. This is plausible since snacking and sweet food consumption by children and adolescents has been associated with parental/caregiver consumption of these types of foods [24, 25]. Indeed, since the caregiver is largely in control of the food environment in the years before going to school, the liking for, and consumption of energy-dense/nutrient-poor snacks will occur in environments where those foods are readily available [26].

Among the preschool children, snacking away from home was significantly more common among children from households with higher education and income, but this was not a consistent finding across age-groups. Preschool children with caregivers educated to graduate-school level were more likely to snack away from home. Preschool children in households from lower education and income groups demonstrated a higher prevalence of snacking at home. Little is known about the influence of socio-economic status and/or education level on snacking location in very young children. A European study found that maternal education level was inversely related to preschool children’s intakes of snacks and sweet beverages [27]. Since caregivers with a higher education level may be more likely to work, there is the potential for confounding effects of education by work status and this should be investigated in future analysis. This highlights the potential importance of both the home food-environment in the case of lower-education/lower-income households and snacking behaviors, and the child-care environment in the case of higher income/higher education/working households. Such insights help to delineate the roots of the snacking behaviors and highlights opportunities for intervention.

By 12 months, approximately two-thirds of children consumed snacks at home and one third away from home, indicating that dietary behaviors related to the consumption of snacks outside the home starts at an early age. Early dietary shifts towards the consumption of calories outside of the home parallels that observed among older American children and adults who consume approximately two thirds of calories at home and one third away from home [28]. However, the mean contribution to total energy (kcal/day) provided from snacking at home was lower across all age groups, with more energy consumed from snacks when they occurred outside the home.

In terms of the location of out-of-home snacks, the daycare becomes an increasingly important location for snack consumption among older children versus younger children. This possibly reflects the primary caregiver undertaking employment and reliance on daycare by working families before children start school. The daycare setting was one of the most frequently reported away-from-home snacking locations, with 37% of all out-of-home snacks being reported as consumed at daycare (data not shown). Restaurants, cafeterias, fast-food, delis, take-out, the store and other locations contribute to the remaining 63% of out-of-home snacking occasions (data not shown).

A recent qualitative study in the US highlighted that location emerges as a qualitative theme in the discussion of snacking with caregivers of preschool children [29]. In this study, caregivers talked about snacking in relation to it occurring inside or outside the home. Inside the home, the snack location was usually mobile, occurring anywhere in the house, whereas the meal occasion was usually sitting down at the table. The authors discuss how the caregiver conceptualization of what constitutes a snack may be coupled to the location in which it occurs. A particular food eaten in a particular location, at a particular time, may be considered a snack, however, the same food, consumed at a different location, at a different time, may not be conceptualized as a snack by the caregiver. This may indicate that the context in which snacks are consumed may determine if caregivers perceive a food as a snack or not.

Even though small children may need more frequent (smaller) meals and snacks, there is little room in the diet for calorie-dense, nutrient-poor choices [30]. We found that the snack foods provided to children, on the day of the survey, deteriorated in the older children versus the younger children as caregivers gravitate towards sweets/sweetened beverages among the > 2 years age group. Indeed, the sweets/desserts and sweetened beverages were the most frequently consumed snack foods among children aged > 2 years of age, and were consumed by 60–83% of children both at home and away from home. The snack foods consumed on the day of the survey, therefore, appear to be less nutritious in the older children, versus the younger children. This is a concern for the overall diet quality of these young preschoolers since they may adopt these unhealthy behaviors which may persist into later childhood and adulthood [31, 32].

Snacking behaviors among infants and young children differ according to the time of day, both in terms of the percentage of children consuming snacks and the energy contribution from morning, afternoon and evening snacking periods [2, 17, 33]. We showed how snacking behaviors varied among infants, toddlers and preschool children according to the snacking period. The most popular snacking period among all age groups, at home, was the evening snacking period, whereas the most popular snacking period away from home was during the afternoon. A deeper analysis of the reasons behind offering snack foods, at different times of the day, is required, and may help support the evolution of dietary guidelines about snacking along with education/intervention to improve snacking choices for very young children. Models of food choice behavior suggest that eating episodes such as snacking may have a cultural classification [34]. Indeed, qualitative studies indicate differences between U.S. and European attitudes towards snacking, with the feeding of children in-between meals at specific clock times featuring in French and Swiss cultures, but not in the U.S. [35] Geographical differences are apparent in terms of snack intake behavior and there are clear variations in the way dietary guidelines describe snacking recommendations around the world [36–40]. Future analyses may wish to evaluate how snacking behaviors vary according to geography and race/ethnicity.

Whilst the FITS survey methodology provides valuable data on dietary intake in the first years of life, it is important to acknowledge certain study limitations. Participants may have over-reported consumption of healthier snacks and under-reported the consumption of less healthy snack-food items (social desirability bias). The success of 24-h recall depends on the participant’s ability to remember, communicate and judge portion sizes, which may prove difficult in certain contexts such as the recall of snacks eaten in the daycare environment. The definition of a snack episode was self-defined by the caregiver and there is controversy around the definition of the terms “snack” and “snacking” and the subsequent interpretation of dietary intake data related to these terms [41]. In addition, length/height and weight measurements are not taken in the FITS study, rather, self-reported data on length/height and weight is collected from the caregiver. Therefore, analysis of BMI and its association with dietary behaviors is not possible.

This paper provides several important insights for those involved in research and nutrition policy or practice. First, at-home and away-from-home snacking behaviors are not the same and the behaviors are different in older children versus younger children. Secondly, the food choice of snacks become less nutritious as children approach 2 years of age. This highlights an opportunity for future dietary guidelines to discuss snacking choices, including the location and timing of snack provision for infants, toddlers and preschool children. Reducing the snack consumption that occurs from sweet foods and beverages should be a priority, particularly in children aged 12–47.9 months, and directing parents and caregivers towards nutrient-dense, affordable snack-choices is advisable. Young children, who will naturally express hunger between meals, may present a dilemma for the caregiver, therefore the importance of anticipation and preparation for in-between meal hunger should be a priority for education about eating in between meals. Further research should be conducted in this age group to better understand snacking behaviors according to socio-demographic factors and how snacking episodes contribute to nutrient intakes.

## Conclusion

Data on dietary patterns, including how location may influence snacking behaviors, can make an important contribution to the development of pediatric feeding guidelines and potentially contribute to dietary guidelines for the population from birth to 24 months in the United States which are due to be released in 2020 [42]. Dietary guidelines about snacking should emphasize limiting sweetened beverages and energy-dense, low-nutrient foods such as cakes, pies, cookies and pastries as snack choices for very young children. Future dietary guidelines for the birth-24 month age-group may wish to raise the importance of the location of snack consumption and highlight the importance of education of child-care providers and caregivers about age-appropriate, nutrient dense snacks that might be provided in the child-care setting. Indeed, further analysis on the food consumption of children attending daycare, using FITS data, is warranted. In addition such data can highlight opportunities for education or interventions aimed at improving caregiver-driven snacking behaviors among infants, toddlers and preschool children. It is important that parents be educated about the choice of snack food for their young child, both at home, and away from home. In addition, guidance should be given to parents and caregivers related to anticipating young children’s snack requirements and how to provide nutrient-rich, snack-food items (such as fruits, vegetables, whole grain breads and cereals, lean proteins and dairy products), particularly when away from home.

## Abbreviations

FITS: The feeding infants and toddlers study
NDS-R: Nutrition data system for research
NHANES: National health and nutrition examination survey
SAS: Statistical analysis system
SE: Standard error
U.S.: United States
